# Getting Ours? “Girlbossing” and the Ethics of Nurse Reimbursement Models

**DOI:** 10.1089/heq.2024.0059

**Published:** 2024-07-26

**Authors:** Danisha Jenkins, Jennifer Cohen, Rae Walker, Patrick McMurray, Jess Dillard Wright

**Affiliations:** ^1^ School of Nursing, San Diego State University, San Diego, California, USA.; ^2^ Department of Global and Intercultural Studies, Miami University, Oxford, Ohio, USA.; ^3^ Elaine Marieb College of Nursing, UMass Amherst, Amherst, Massachusetts, USA.; ^4^ School of Nursing, University of North Carolina, Cary, North Carolina, USA.

**Keywords:** reimbursement, capitalism, nursing industrial complex, nurse reimbursement

## Abstract

**Introduction:**

This article politicizes a reimbursement model proposed by some professional nursing associations that aim to better align the price of nursing labor (nurses’ pay) to the value of nursing and make nurses’ contributions more visible.

**Methods:**

Using the concept of “missing care,” the critique reveals how professionalization directs attention to individual-level interactions between care seekers and practitioners while obscuring from view the harm inflicted by social institutions and structures constitutive of a capitalist political economy and the related carceral state.

**Results:**

Direct reimbursement models render practitioners complicit in the harms perpetrated and perpetuated by the health care industrial complex while professionalization processes are deployed to reduce cognitive dissonance (and moral injury) produced by combining harm with nursing’s normative principles.

**Discussion:**

We describe and trace the complementary capitalist imperatives of extraction-based profit maximization and efficiency through the health care industrial complex to demonstrate how formative those imperatives are of the health care system, care-seekers’ outcomes, nurses’ experiences, nonconsensual modes of data collection, and surveillance.

**Conclusion:**

The naturalization of racial capitalism and the precarity and violence it entails foreclose the creation of ethical alternatives that prioritize well-being instead of the pursuit of profit that could bring the provision of and payment for care closer to the normative principles held by practitioners.

## Introduction

How nurses demonstrate their value is a perennial question, predicated on decades of cyclical workforce shortages and health care crises assumed to revolve around the disconnect between the *price* of nurses’ labor—nurse pay—and the *value* of nursing in a context of long-term disinvestment in health care in the United States. The question of nursing’s value gained urgency during the COVID-19 pandemic and remains central as the health care-industrial complex (HIC) implements a new post-COVID business-as-usual. This reality was reflected in discussions of workforce pressures and reimbursement models at the 2023 International Council of Nurses (ICN) Congress.^1^ Likewise, nursing’s value is a central concern for the American Nurses Association (ANA): the organization is promoting payment models to “expand nursing practice and elevate the value of nursing through direct reimbursement for nursing care delivery, management, and coordination outcomes.”^2^ These arguments conflate value with price and thus seek a reimbursement model that better aligns the two. However, prices often fail to reflect *value* in capitalist economies. For example, despite its central importance to maintaining and upholding the US economy, pay for childcare, eldercare, teachers, and other kinds of care provision remains low despite its high value.

On its face, demanding nursing’s worth in the existing economic system is a powerful reformist “girlboss” project that elevates nursing by putting health care money where care happens. Superficially this appears uncomplicated, but digging deeper into the harms of capitalism points to the harms of the HIC. In practice, these efforts discipline nursing to the assemblage of the health care industrial complex, predicated on ideologies and logics of capitalist political economies of care. This, as we will demonstrate, undermines the ethos and praxis of nursing by producing and reproducing systems that harm people, communities, environments, climates, countries, and worlds. The incommensurability of a capitalist political economy and the health and well-being of all people must be at the forefront of conversations about nurse reimbursements, even as we endeavor to shift perceptions of nurse value.

This article examines the ways in which the push for billable reimbursement models for nursing care like those advocated by the Commission for Nurse Reimbursement^3^ is—at best—a confused and problematic reformist approach that subordinates nurses to still more productivist metrics. Because nursing is a large and diverse profession characterized by considerable epistemic plurality, this may not be a problem for all nurses. However, we consider what is at stake in trying to exact nursing’s pound of flesh from the health care “system” as it presently exists, a move that shores up structural inequities that enhance capital accumulation.^4^ Given what nurses profess in our Code of Ethics, it is hard to imagine that an argument that implicitly—and explicitly in some instances—asserts racial capitalism could be the rallying cry. Instead, we demand transformative alternatives to a system that cannot be redeemed. Ultimately, we arrive at the conclusion that “girlboss” reformation of reimbursement models is not an example of justice-oriented “activism.” Rather, it is evidence of nursing’s professional failure to recognize who nurses are, with whom nurses align, who and what nurses struggle with, and which systems and structures degrade nursing’s imagination of the future.

## Where to Begin?

As we begin our analysis of efforts to devise a nursing reimbursement model that appropriately values nursing, it is first necessary to position ourselves. We are nurses and a feminist political economist. Our nursing work is informed by personal experiences of delivering care in health care systems set up to exclude those folks who are deemed surplus.^5,6^ Our work, education, and lived experience are rooted in the colonial empire of the United States, which has an indelible impact on what we know, how we think, and what it means to be. We have occupied and continue to occupy positions of power and have been complicit with the harms of which we are so critical. We do not have all the answers. In fact, we do not have many answers. We do have questions. We are learning and unlearning every day. When approaching solutions toward economic problems, we are generally guided by the questions, “Who does this serve? Who does this harm?” and animated by a commitment to and the imagination for a world in which we achieve all care for all people, informed by our commitment to ethical care. We now consider those who seek care and the impact of capitalist political economies on their care realities.

### Seeking care

Considerable attention is paid, in the nursing literature, to the phenomenon of “missed care.”^7–9^ This kind of literature relies on counted and quantified nursing care events, instances where care could either be delivered or not, counted as done or not done. Such a construction presupposes that care can be counted, discretized, and isolated from the broader systems that constitute care, which is a precarious proposition for a practice discipline that positions itself as holistic and committed to the well-being of society.^10^ This kind of focus on care within the boundaries of clinical spaces, which in some respects makes sense as these physical spaces render clear boundaries, aligns with the neoliberal project of privatization, extraction, and capital consolidation. However, keeping the missed care gaze fixed on the individual nurse and care recipient keeps the focus narrow when the scale is much larger.

Taking a broader view, moving into a different level of abstraction points to the folks who are missing from care as a function of the care systems and structures themselves.^6^ Here, consider the value judgments assigned to folks by the HIC, where heads in beds are not created equal, stratified by payor type. Folks who cannot pay constitute *surplus*, “a collective of those who fall outside of the normative principles for which state policies are designed, as well as those who are excluded from the attendant entitlements of capital” (p. 22), according to *Health Communism* authors Beatrice Adler-Bolton and Artie Vierkant.^5^ People who cannot pay—a growing slice of the US populace—do not receive care.^11^ They are missing and may grow less well the longer care is delayed, putting more distance between the care seeker and the “normative principles” Adler-Bolton and Vierkant point to as essential under capitalist regimes. According to this logic, as people become ill, they become less deserving of capital’s “attendant entitlements,” inclusive of health care. The reality is that, in the necropolitical economies of health care in the United States, the power to determine who lives and who must die is stratified according to the priorities of for-profit health care entities.^12–15^ Health care is rationed, not according to the death panels anticipated by opposition to the Affordable Care Act, but rather by the pernicious and invisiblized thanatopolitics of capitalism. Missing care indeed.

Care might also be missed because people actively avoid care. Despite the profusion of rhetoric on well-meaning, goodwill, and virtue in nursing and adjacent spaces, care is not generative or affirming for many. Examples of this proliferate, both historical and contemporary. Consider the pathologization of queer sexualities and gender identities, which at one time were listed as disorders in texts like the *Diagnostic and Statistical Manual*, a product of biomedicine’s compulsory heterosexuality and cisnormativity.^16^ Refusing to acknowledge queer families in acute care settings harmed people. Practices such as conversion therapy harmed people. Refusal to affirm gender identity harms people. Health care can itself be harmful, violent, and painful. This harm, this violence, and this pain are not just located in the interpersonal interactions of those seeking care. Instead, the assemblage of health care is a site for the production, dissemination, and reproduction of white and cisheteropatriarchal normative principles.^17–19^ Looking no further than the pervasive discourse of evidence-based practice (EBP)—which *is* a discourse, which *has* assumptions, and which *is* fallible despite the dogmatic reverence conferred by the health sciences—the priorities of white, paternalist hegemonies are apparent. The evidence base upon which is predicated is developed by white empiricist hegemonies that take for granted who and what is worthy of study as well as who is permitted to study and who can claim epistemic authority.^20,21^ Compounding the problematics of EBP, health monitoring technologies also encode inequity, evinced by recent findings that pulse oximetry algorithms fail to detect hypoxemia in folks with darker skin tones as readily as with their light-skinned peers, a single example among many.^22^ Building from such precarious technologies generates an evidence base that elides its own shortcomings which are in turn enshrined in standards of care that harm.

Folks also avoid care because of the carceral implications of care.^23,24^ People with substance use disorder may avoid prenatal care upon learning they are pregnant to avoid entanglement with the justice system, entanglements that could result in incarceration, removal of their child at birth, or long-term engagement with the child welfare system.^25,26^ Parents experiencing poverty strategically navigate institutional engagement with their children to limit their exposure to so-called welfare and justice agencies.^27^ Fearing deportation and other immigration-related consequences, adults in migration also avoid care, delaying essential care that leads to excess morbidity and mortality.^24,28^ The collusion of the HIC with other carceral systems is extensive, co-constitutive, and even. Entry into health care can and does present considerable risk to many, a risk that is amplified for people who occupy multiple marginalized social locations.^23^ Care, then, is fraught for many who seek it, and this precarity is further reinscribed by the invisibilization of nonindividualist, structural, and institutional sources of “missed care” upon which the proposed reimbursement model is built.

### Giving care?

From the perspective of the caregiver, there are many parallel considerations. While we are speaking specifically about nursing as a caregiver, we recognize that professionalized nurses are only one subset of people who can and do provide nursing care. We further recognize that the project of nurse professionalization is rife with elitism, racism, sexism, ableism, cisheteronormativity, and so on *ad nauseum* inherent, essential to the project of constructing the normative principles by which distance is created between carer and care-recipient. Marking out this distance enables professionalization, territorializing the concept “nurse,” and through nursing care, territorializing “patient” simultaneously.^29–31^Most recently in the United States, professionalism demands conformity to standards, expectations, and norms associated with corporate stakeholders and institutions, a central conceit of this article. This is connected with the political economies of health care in the United States, a topic that receives considerable attention below.

Nursing’s professional legitimacy in the current configuration of US health care is contingent on maintaining cordial relations with medicine, echoing the family dynamics articulated by feminist nurse firebrand Joann Ashley.^32^ Describing what she called the “hospital family,” Ashley’s^32^ critique highlighted the paternalist qualities of medicine in health care, exercising oppressive control over nurses and the people in their care. While there are critiques to make of Ashley’s analysis, this configuration does evoke considerations of reproductive labor and social reproduction, the feminized work of life.^33,34^ Nursing can be understood as waged reproductive labor that attends to the “real” business of health care: medicine. Some will object to this characterization, even asserting that national nurse identifiers and new reimbursement models are designed to address exactly this gap. To the naysayers, we might point to the ways in which nursing care is rolled into room charges, literally part of the furniture.^35^ To the problem-solvers, we reserve most of our remarks for later in the article, but note that discretizing nursing work and accounting for its value in the vernacular of economic production presents a considerable existential threat to the discipline we know and love-and critically to those who need nursing care.

In the gendered world of the hospital family, nursing does much of the reproductive labor that makes productive health care labor possible.^32,33^ The reality is that nursing makes hospitals go. Nursing makes health care systems go. Evidence of this might be found in the specter of nursing strikes. Nursing strikes are controversial. Some nurses perceive striking as an abrogation of nursing’s obligation to care, a moral failure, and even crass.^36,37^ This, in our view, is naive and hazards exploitation, rooted in self-sacrificial, maternal tropes of the profession that fail to reckon with the realities of working within a capitalist enclosure. Nurses are not angels nor saints nor everyone’s mothers and cannot sustain the work of nursing on goodwill alone. Nursing strikes are ruptures, peeling up the edges where the invisibilized work of nursing—the fundamental *a priori* to productive labor in health care settings—becomes perceptible. The value of nursing’s reproductive labor is legible in strikes because of the disruption it causes.

Nursing is in the business of getting stuff done under any circumstances. Making it work. This, we contend, is a remarkable feature of nursing, a testament to the resilience and ingenuity of our peers and comrades. It is also one of our most profound vulnerabilities. Under the managerial logics of capitalism and its attendant metrics, it is very difficult to make the case that something is not working if nurses are able to make things go. Even if that is at great expense to individual nurses and just by the skin of their teeth. Things are still functioning, at least according to people whose voices currently count. This logic means pleas to rationality, care, ethics, beliefs, andanything other than the bottom line are immaterial. This is a real problem for the embodied, relational, worldbuilding human praxis of nursing because this work is not standardized, reducible, or discretizable. From our perspective, it seems clear that nursing in the enclosure of health care in the United States is not working—not for the people nurses care for nor for nurses themselves. The proposed reimbursement model puts further distance between the realities of care and what we espouse as our “normative principles.” This leaves a lingering question: who does this system work for?

## Capitalism 101

As they are presently constructed, health care assemblages gobble up the spoils of the systems, institutions, and structures that create the realities we outlined in the sections earlier. And nurses are ill-prepared to navigate: nursing curricula invest little in educating aspiring nurses on issues related to health care finance and economics. Even less attention is paid to critical analysis of health care’s position within the broader economy and political systems. The nurse reimbursement reform initiative is demonstrative of the tendency to see our current economic system as naturalized, immutable, and the only way. The question of *value* in the marketplace presupposes neoliberalism, scarcity, competition, and commodification of people and care. Because the conversation about nurse *value* is entangled in the web of capitalism, we find it necessary to provide a contextual foundation of capitalism as a political economy. We begin with a brief discussion of value and profit maximization, then turn to the broader view.

### On value and profit maximization

In the conversations around nurse reimbursement and nursing labor, value is frequently left undefined but there are at least three definitions at play in our considerations here: value-added, perceived value, and amorphous value. Value-added accounts for the value contributed to a given situation by various elements and interlocutors while perceived value attends to contribution and status; amorphous value resists quantification but remains an imperative part of nursing care, life affective labor. In this imaginary, value is recordable, quantifiable, and billable. This definition underlies the arguments for the new reimbursement model. But price is not just conflated with value, price dictates value according to at least one professional organization, as we will see from the American Nurses Foundation. In this formulation, value means something more than price and might be understood as akin to perceived contribution and perceived status. This framing inverts mainstream economic thought, in which value determines payment. In practice, the proposed reimbursement model is grounded in the mainstream economic perspective: through quantification, value becomes the “value-added” generated by nursing in the production of health care. This is an economistic, “bottom-line” conversion that uses universal nurse identifiers to track nurses and their labor. The model therefore does not accomplish its supporters’ intended outcome, at least not directly. Instead, it forces causality into a market-based framework through quantification-as-method. Hence, in the reimbursement model value-added dictates payment which supporters hope defines perceived contribution and perceived status.

Supporters may not find this economism problematic, but it is the economism that renders the model incapable of capturing the real value of nursing, much of which is not quantifiable. One reason the model fails is that it is necessarily incomplete because nursing labor is integrated in almost every aspect of health care. A hospital can bill insurance for a bedpan and a catheter, but those items (inputs to health care) do nothing without the nursing labor that puts them into action. The reimbursement model aims to capture the labor of putting them into action but fails to capture the affective labor that work requires and the amorphous value it creates in the forms of patient comfort, well-being, dignity, safety, perceived support, and just care, all of which are probably closely related to patient outcomes.

In a capitalist political economy, the profit motive is the engine that drives capital accumulation, or economic growth. The profit motive also drives investment in health care (and disinvestment in informal caregiving, community health, public health, population health, and global health). For clarity, it is noteworthy that at the micro-level a sizable proportion of the health care providers that employ practitioners are nonprofit entities, meaning that they are revenue-maximizing rather than profit-maximizing. Although these motives differ in theory, nonprofit organizations, especially in health care and education, have adopted business models that make them almost indistinguishable with the exception that nonprofit organizations do not pay taxes. Hence, we do not distinguish for-profit from not-for-profit, nor revenue maximization from profit maximization in our analysis even as we see acceptance of capitalist priorities to extract value from labor through discourses of “efficiency” and doing more with less.

### Political economies and health care

Political economy is the interaction between the market, state, and society, pointing to the social and political relations of economics.^38^ Political economies describe how economic policies and practices both influence and are influenced by political factors and how they in turn shape resource distribution, opportunities, and ultimately societal power. A political economy situates economic systems that are deeply intertwined in social and political processes and institutions. The concept of political economy and the ensuing realities that unfold as part of them demonstrate the ways that budgets are moral documents, allocating resources according to political, social, and economic priority. Political economies discipline people in striking ways. The health care system is critical as a political economy because it is a powerful mechanism of labor discipline. The employer-based health insurance system combined with increasingly inaccessible health care outside of the private market potentiates control over populations and keeps workers attached to their jobs. In addition, although full-time employment is the most reliable way to obtain comprehensive health benefits, the number and types of jobs that offer full benefits are falling and changing. Positions with the most comprehensive and robust health benefit packages tend to be those of higher leadership, higher pay, and requiring higher credentials. This further entrenchment of health care in capitalism both reproduces and maintains systems of oppression and discrimination.^39^

The US health care system is a tremendous place of employment, site of investment, and arena of consumption. Because of this, the draw toward exploring the *business* of nursing under capitalism is not surprising.^39^ Health care is a desirable breeding ground for capitalist penetrations in ideology and practice. Health care-and therefore nursing is potentially and actually extremely profitable and revenue-generating; the market is captive, it employs a powerful sense of control over the public, the state is a guarantor of profit, and it allows exploitive institutions to project a false image of conspicuous benevolence.^40^

### What is capitalism?

McKinlay described that the prerequisite and defining characteristic of capitalism is the inexorable predatory requirement of profitability, “the act of invading, exploiting, and ultimately despoiling a field of endeavor—with no necessary humane commitment to it—in order to seize and carry away an acceptable level of profit.”^40^. Capitalism depends on wage labor in which workers sell their labor to employers in exchange for a wage. Capitalism perpetuates economic inequality because those who own and control the capital seek to accumulate wealth and power. Capitalism derives value in people or things by turning them into a commodity; something that can be bought and sold for profit. This includes objects, natural resources, and increasing aspects of human life and survival, like health care.^41^ Under capitalism, such vital decisions on the allocation of resources, types and locations of emerging technologies, and investments in manpower are made following the requirement of profitability. Because of the very nature of capitalism, the most socially needed activities cannot be measured according to profitability criteria.

In systems where capitalism is deeply rooted, Marx purported that potentialities other than capitalism—especially when words like “imperialism” and “class struggle” are used—are quickly dismissed,^41^ framed as immutable and eternal. Capitalism justifies and rationalizes limiting efforts for collective benefit, including health.^42^ Simultaneously, the rhetorics of capitalism remain generally unquestioned despite a capitalist political economy’s inability to meet the needs of all of us. This is true especially in nursing, when our professional code of ethics asserts health care as a basic human right, while capitalism instead promotes restricting health care to most and denying it altogether to some. Despite persistent and prevailing empirical evidence that the activities of the HIC run in violent opposition to social and community needs, capitalism and health care continue to be entrenched as a political economy.

### Disaster capitalism

Although it is tempting to think of crises, disasters, and catastrophes as somehow exceptional, under the logic of capitalism disaster states are used to recalibrate baselines.^43^ Austerity measures are invoked in the name of coping with an emergent situation, the promise of a return to pre-crisis circumstances implicit. However, as the crisis recedes, the unthinkable provisions of disaster demonstrate just how readily systems and workers adapt to spartan conditions and baselines never recalibrate to previous norms. In the political economy of capitalism, these crises stack up and “come to define our ways of living, being, doing, scraping, scrimping, scrambling, characteristic of our past/present, deterministic of our present/future.”^44^ The priorities of capitalist political economy as social relation leave burnt-over infrastructure in its wake,^38^ the next disaster washing over eroded systems, structures, institutions, and communities, flooding an already-waterlogged terrain.^43^ This kind of rationing, though, is applied unevenly, the brunt of the austerity borne by workers and care-seekers.

The realities of health care in the COVID-19 pandemic provide a ready and compelling case study for thinking through the logic of disaster capitalism. Well before the pandemic—and in cyclical fashion since the professionalization of the discipline—the nursing workforce was grappling with burnout and staffing shortages, an unsteady foundation.^45–51^ The care demands of the pandemic shook crevices and cracks into caverns and fjords, nurses worked under dangerous conditions with mandatory overtime, often without the protections of appropriate personal protective equipment (PPE).^44^ Nurses’ outcries were met with nightly rounds of applause, at least for a while,^52^ censure for fundraising to procure PPE,^53^ and refusal to invoke the Defense Production Act by the Federal Government.^54^ Over time, as nurses left abject working conditions and travel nurses took contracts that assured appropriate pay, the clapping receded while hospitals and insurance companies posted record profits.^55–59^ Disaster capitalism manufactures new normals and propagates inequities in the name of extraction.

### Racial capitalism and the health care industrial complex

The capitalist economic system in the US is one that was built on stolen land using the forced labor of stolen people. Cedric Robinson described that capitalism was born of racialized societies, providing a structure for racist establishments today (such as the HIC), that disproportionately kill racialized people.^60^ The bedrock of the US economy was scaffolded violently using chattel slavery; a system in which Black humans were seen as valuable only insofar as they produced profit. The racial capitalist infrastructure of the US grows via the destruction and depletion of resources; a process that consistently leads to inequities, trauma, poor health, and death for people of color and most significantly for Black and Indigenous people.^61^ Robinson asserted that the development, organization, and expansion of capitalist society pursued essentially racial directions. The modern US health care system grossly demonstrates that racialized exploitation and capital accumulation are mutually enforced phenomena.^60^

The racialization of capitalism is a violent project. Marx also introduced the significance of exploitative means of capitalist economics, writing “In actual history, it is a notorious fact that conquest, enslavement, robbery, murder, in short, force, play the greatest part” (p. 875).^41^ Employing these methods, following Marx, demands a human hierarchy predicated on the assumption that humans can be divided into superior and inferior groups. This is operationalized by the invention, production, and codification of race. Once established, these are the same racialized assumptions that give rise to the conditions required for slavery, colonization, and genocide, and provide that “[…] subjection of those whom capital expropriates is a condition of possibility for the freedom of those whom it exploits”.^62^ Capitalism is predicated on racist violence. Health care, nursing, is not exempt.

The totalizing stronghold of racial capitalism as the underpinning of the US health care system clouds nursing’s imagination for any other potentialities.^63^ The extraction of labor power from social relations and sucking it up into the gears of the capitalist machine is a historical foundation of capitalism, and racialized expropriation becomes increasingly severe as less tangible resources become available.^64^ In the post-industrial-revolution period humans and their ascribed values have been hierarchized in sexualized and racialized ways that potentiate their exploitation, oppression, and disposal. Racial capitalism is a technology of antirelationality that dissuades us from considering noncapitalist and nonstate functions as possible;^65^ any other prospects for the future are rendered futile. As an extractive system that exerts violence, racial capitalism exerts social separateness, and in order to function requires the disjoining of relations between other humans and nature. The proposed reforms in reimbursement models strive to overcome the ways in which nursing is cast aside, isolated, oppressed, and devalued under the auspices of capitalism. And yet, the proposal is a reinvestment in racial capitalism (and therefore colonialism and white supremacy), and thus a self-defeating strategy. Success and making it to the top is only possible when we push people to the bottom.^66^ We ask why we are so apt to further hand over the precious social relations of nursing to a globally violent system of extraction that does so much harm.

## White Feminist “Solutions” for Racial Capitalist Extraction

From our bathroom breaks to our sleep schedules to our emotional availability, Millennials are growing up highly attuned to the needs of capital markets … Efficiency is our existential purpose, and we are a generation of finely honed tools, crafted from embryos to be lean, mean production machines.^67^

Neoliberal white feminism has naturalized capitalism and perpetuated the myth of individual exceptionalism, a manifestation that Reich^68^ describes as *wealth supremacists.* Neoliberal white feminists are “[…] less concerned about solidarity and thrive on the glorification of economic success of the fourth industrial revolution.”^69^ This brand of feminism finds solutions in the market economy to address the problem of advancing (white) women in the corporate sphere. These #girlbosses see success in women having an equal number of seats at the table to enjoy the luxurious capitalist feast, no matter how toxic and violent the spoils. No matter who goes hungry. The #girlboss is described by Alexandersson and Kalonaityte as a framing of feminism that “treats equality as an already accomplished fact in Western societies, assuming that women need to take an enterprising approach in order to succeed in any—or every—area of their life” (p. 419).^70^ The girlboss ideology is built on the contradictory premise that someone’s success depends on their assertiveness, sense of empowerment, and “sleep when I’m dead” mentality while simultaneously being calm, practicing self-care, and not working too hard. Girlbossing is an ideological process in which an entrepreneurial spirit is celebrated to “get ours,” reducing the person in question to a tool of capitalist production, their value commensurate only with their ability to produce and consume. This, according to Harris,^67^ is particularly powerful in the femme millennial workforce that is in a constant state of proving their value; striving to work hard, play hard, and make good on the promise of capitalism to become industriously self-reliant. This gives rise to the invisibilization of exploitation and labor relations, replacing it with a white feminist entrepreneurial ideology.

In the conversation of value, particularly for a white female-dominated profession, we must note the intersectionality of this dialogue. This conversation is an opportunity to appreciate that “getting ours” while not attending to the racist functions of capitalism only serves to subjugate our collective selves further, although this function is unevenly distributed. The assigned categories of difference as well as their meanings and assumed value include hierarchies of the gender binary and are produced by expropriation.^71^ This is especially evident in the extraction of care labor in which women internalize (or are professionally ascribed) a morally laden duty to care. These extractive, differentiating, and hierarchizing logics are inherent to the very essence of capitalism. Wang further asserted that “while it could be said that *disposability* is the logic that corresponds to racialized expropriation, gendered subjectivation has its corollary *rapeability* [.…] these expropriative logics are not mutually exclusive, as women who are not racialized as white and gender-nonconforming people may be subject to a different set of expropriative logics than white women” (p. 120). The reality is that the proposed reimbursement model is predicated on acceptance of the oppressions and inequities capitalism demands to maintain and enhance profitability.

## Implications of Capitalist Political Economy for Health Care

Unfettered capitalist futures are bleak. We are spiraling toward, maybe even living in, times of unprecedented death accompanied by increases in chronic disease, mental illness, suicidality, environmental catastrophe, mass malnourishment, famine, and war: an empire of poverty and suffering.^42^ Although there is ample literature that makes visible (at least statistically), the mass death that surrounds us, it seems our profession, and particularly nursing professional organizations, choose to look through it, “[…] we look through human suffering and see security and civil peace. Death is our atmosphere, too close for us to see, and too much a part of us for us to comfortably reckon its work in our vital economies.”^15^ We are at a powerful juncture, in which the profession of nursing must determine and articulate the ideology of the political economy we propose for the future. A capitalist political economy and meeting the needs of the community, are diametrically opposed. McKinlay^40^ argues, “Since there is no logical connection between the dictates of profitability and the fulfillment of collective needs, one cannot assume, as many do, that these two will naturally and inevitably be joined. Both are premised on distinct and often conflicting ideologies. Consequently, medicine under capitalism will never contribute to improvements in health unless such improvements facilitate an acceptable level of profit” (p. xiii). It is clear we languish for leaders who assess our value and commit to getting the proverbial seat at the table. If we instead look to those made dead and left behind by this capitalist health care system, we can begin to see the senselessness and irrelevance of the state and current order.^15^

Reimbursement shapes the care we provide. Under a system designed for profit, this has historically meant commodification of human survival and a stringent focus on technological fixes that are easily billable, but not effective.^72^ There is significant concern with how nursing practice is conducted under the constraining contexts of capitalism. In its current state, we know that nursing practice is already structured by profitability and productivity, increasingly more so under the ever-panoptic eye of the electronic health record.^63^ Further investment in the political economy of capitalism means further investment in a practice dictated by increasing economic value; a value drawn on extraction, efficiency, productivity, technology, and economies of scale. The incommensurability of a capitalist health care economy and the health and well-being of all people must be at the forefront of conversations on nurse reimbursement, despite hopes for a shift in perceptions of nurse value.

## Capturing Nursing Value

Up to this point, we have been explicating theoretical foundations, building vocabulary for analyzing and understanding nursing work, labor, and value in health care systems. We are now transitioning to a more direct discussion of the matter at hand: nurse reimbursement. Asserting that “payment defines value,” (para. 1) the American Nurses Foundation^35^—the philanthropic arm of the American Nurses Association—embraces a #girlboss approach to playing the capitalist game predicated on the assumption that the “lack of reimbursement to nurses for the care they provide hides the value of the role they play in integrating quality, safety, and efficiency” (para. 1) gives rise to the invisibility of nursing work. The remedy, according to the American Nurses Association, lies in creating universal nurse identifiers (UNI), unique codes issued to individual nurses with the intent to “quantify nursing-sensitive outcomes and increase the evidence of their contributions to patient care.”^73–75^ Described as a fingerprint to enhance nurse visibility, the UNI is a defined code that represents one nurse in health care technology and can identify them across health care organizations.^74^ Describing the challenge of quantifying nurse-sensitive outcomes, proponents of the UNI state that the identifier will provide tangible evidence of how nursing care affects patient outcomes.^74^ When describing the necessity of UNI, the Nursing Knowledge Big Data Science Conference Health IT Policy Workgroup lists nurses and employers tracking nursing licensure across job and location changes as the top reason.^76^ Chan et al. (2023) assert the value of UNI in researching workforce trends. Although nurses already have what could be understood as a UNI assigned when applying for licensure, adoption in electronic health records has not been standardized.^75^

This reliance on UNI—and particularly its connection to “reimbursable” care—is demonstrative of the nursing profession’s continued and belabored effort to explain and quantify phenomena of nursing that are not directly measurable and empirical. This positivist reduction of nursing care to objectified interventions impedes our ability to grasp the meaning and value of nursing in its inexhaustible variations, meaning, and culture, which are also part of the essence of nursing as a phenomenon. This method and limited purview invite oversimplification of a phenomenon by eliminating the less tangible aspects of complex care provision from nursing. At this point, the reduction is so disassociated from the phenomenon that it risks becoming entirely unusable to the people that it was designed for, and it offers a picture of unreality.^77^ In the section that follows, we will first examine past efforts at making nursing work legible in the marketplace of health care and then consider what is currently on offer.

### National nurse identifiers

The Alliance for Nursing Informatics (ANI) recently endorsed the implementation of a UNI, modeled after identifiers used by physicians and other providers who bill for services. ANI envisions that UNI would follow nurses across the course of their careers, tracking individual nurses’ actions and care contributions, and recording those contributions in electronic health records as well as other platforms such as enterprise resource planning systems and various health IT vendor platforms. Nursing professional organizations have put forth two distinct proposals for how to operationalize this recommendation: to have nurses register with the existing national provider identification system (NPI) currently used by physicians, nurse practitioners, and other prescribers in the United States, or to use unique identifiers available through the National Council of State Boards of Nursing (NCSBN). ANI has endorsed the latter, wherein a “bulk enumeration process” available through the Centers for Medicare and Medicaid Services (CMS) would allow for automated assignment of unique identifiers to individual nurses using the number they were provided when they registered to take the national licensure exam (NCLEX).^78^ This procedure would avoid delays and data gaps associated with requiring individual nurses to register for their own unique identifiers through the NPI system, while simultaneously circumventing nurses’ consent to be tracked this way.

In 2023, Walker and Dillard-Wright evaluated policy proposals for a nationwide implementation of UNI using the Consentful Tech Criteria adapted from Planned Parenthood’s acronym FRIES.^79^ Questions of consent are frequently considered in the context of sexual encounters, the provision of health care, and even research; however, less attention is paid to consent in the context of proliferative technologies. To address this gap, the Consentful Tech Criteria evaluates technologies in terms of various dimensions of consent, which according to the FRIES acronym should be: Freely given; Reversible; Informed; Enthusiastic; and Specific. Current UNI proposals failed each of these criteria. The use of CMS’ “bulk enumeration” process fails the *freely given* criteria, as nurses would not have the ability to consent to the tracking of their data. Such surveillance would not be *reversible*—not only are there no systems in current NPI platforms for providers to “take back” or erase their data, but research on big data increasingly indicates individuals can be easily identified even when personal identifiers are obscured. UNI proposals are not *informed*—not only do individual nurses not necessarily have information or control over how their data are tracked and how such information is applied, but current proposals also fail to address how such information might be weaponized. What will become of nurses found to have provided increasingly politicized forms of care (gender-affirming care, abortion care, medical assistance in dying, etc) when they cross state lines into a region where such care is criminalized? Although some nurses might give enthusiastic consent to such surveillance, others will undoubtedly reject such tracking. Finally, the use of a UNI does not allow for consent to specific applications of one’s data, as registration permits all forms of activity to be tracked en masse, as opposed to nurses having control over how such information is applied. None of the UNI demonstration projects to date reported on individual nurses’ control over the application of data that had been surveilled.^80^

If the ANI proposal is implemented, the NCSBN will control the database where unique nurse identifiers and their associated data are housed. Some have suggested this would be a benefit of the change—allowing NCSBN to commercialize the information in the form of tailored recommendations (or requirements) for additional certifications, continuing education modules, and specialty licensure exams. Voting membership of the NCSBN is composed of representatives of state boards of registration for nursing: politically appointed positions usually controlled by whatever governor is in power. There’s been no discussion in recent policy proposals or the literature as to whether NCSBN, as an organization whose voting membership is subject to whatever politicians are currently in power, is an appropriate entity to ethically govern a database containing identifiers tracking the actions of every registered nurse in the United States. This tracking would occur against the backdrop of an increasingly polarized and fraught political landscape, wherein specific forms of nursing care are increasingly criminalized.

To date, the nurse leaders and authors calling for this change are primarily hospital administrators and informaticists—nurses and other professionals well-positioned to benefit from the implementation of such surveillance. Narratives tend to play on current realities of understaffing and exploitation of direct care nurses: mobilizing popular sentiments and resentment regarding the undervaluing and erasure of care work (“Nurses are still part of room and board!”) to enlist direct care staff in the cause—a cause largely driven by corporate objectives to metricize and “optimize” every last inch of contemporary health care. The primary argument of ANA and associated professional organizations is that nursing needs more data to demonstrate the “value” of nursing care. Ironically, this imperative comes at a time when the value of nursing has just been demonstrated on a global scale—throughout the last several years of this ongoing pandemic. One might argue we already have the data that demonstrates the value of nursing care.

## Other Possible Futures or Is This What We Dream Of?

All the reasons for making a revolution are there. Not one is lacking. The shipwreck of politics, the arrogance of the powerful, the reign of falsehood, the vulgarity of the wealthy, the cataclysms of industry, galloping misery, naked exploitation, ecological apocalypse—we are spared nothing, not even being informed about it all.^81^

The problem with nursing labor in the necropolitical economies of health care in the United States is not that nursing work is unimportant. Nursing labor is a prerequisite for the so-called productive labor of the American HIC. Nursing is the reproductive labor of health care that reproduces, supports, ensures, and nurtures the system and those people in it.^82^ For capitalism to thrive, this kind of invisibilized labor is necessary. In the current capitalist mode of health care, nurses act as sponsors: people who provide capital to producers. Sponsors do not necessarily strive to maximize economic return on the capital they provide but do try to advance their class interests.^83,84^ Care work such as nursing is not production oriented and it does not lend itself to ready quantification and commodification. Impulses to link nurse actions with discretized, reductive metrics to account for their workforces the holistic and professionalized practice of nursing further into an ontological stranglehold.

On its face, to endeavor to quantify the *economic value* of nursing makes sense. After all, nursing is embedded in a capitalist system in which all efforts are toward maximizing profit. The state produces an image that makes us rely on it and for some worship it. To appreciate this dogma, we can understand why some among us cling to its oppression and violence.^66^ The propensity to employ the same tools and tactics of the very system that causes us harm is evidence that the strength of nurses in the fight against asphyxiating political economies relies upon its political consciousness. The collective organizing we must accomplish and the political prowess required to accomplish it requires we release models and approaches that have already failed us. Anderson writes, “Many of us have more in common than we may want to admit, but one of the most important things we share is a threat. This isn’t something to take lightly. State violence is real and deadly. We are largely unprepared and disorganized…We must abandon fantasies that point us toward failed methods and fruitless directions. Things that do not do the work of building revolutionary abolitionist efforts and that favor reformism should be left behind” (PP. 93–94).^66^ This will be a testament to whether or not nursing’s professional ethical codes are truly enculturated.

In this advocacy for transforming nursing reimbursement models, the profession of nursing confronts a recurring paradigm: the presumption that profit-driven solutions will catalyze liberation for nursing as a profession. However, these propositions—advocating for the inclusion of nursing within adjusted reimbursement frameworks—lack robust consideration of what realities they hold up and what realities upon which they foreclose. This obscures potentially, even likely, adverse consequences of such changes on communities who need care. These efforts will be at the expense of some, and extend barriers to health care access. Although nursing may wish to enrich itself, it cannot be done at the expense of people families, communities. In addition, proposed mechanisms for these regulatory models present an increased regulatory burden which is likely to be addressed through novel technological interventions. Such approaches constrain the nursing profession’s intrinsic capacity for agility, adaptation, and innovation in addressing community health care needs and give rise to the question of how might these changes inadvertently impose more significant burdens on the most vulnerable populations. These considerations are essential for those championing this change. Given that nurses should be accountable to the public and communities they serve, the public and communities must be part of every move the discipline makes, ensuring that envisioned reforms enhance health care access and quality without reifying existing inequity and injustice.

It is imperative to think here about whether ethical care is even possible under the auspices of racial capitalism. History shows that sustained change, even in the smallest degrees, requires mass movements that threaten capitalism’s very existence. This is an anathema to leaders and politicians who encourage pragmatic approaches that disaggregate complex problems to address bite-sized downstream issues, leading to a kind of political whack-a-mole.^85^ What is pragmatic or peaceful about continuing, perpetuating, and reinforcing a system that is hurting and killing, nurses and patients alike? The challenges that the nursing profession, health care institutions, and patients face are ruthless failures of the government. We cannot look to the status quo of the state to save us. Given the facts of capitalism and the way in which the nursing profession has been so severely damaged by this very system, we see the investment and efforts of nursing professional organizations to these ends as a betrayal. The Invisible Committee^86^ speaks of this:

And one is always complicit in some way with one’s own betrayal. The fact is painful, so it’s generally denied. We’ve had a blind faith in *crisis*, a faith so blind and so enduring that we didn’t see how the liberal order had made it the centerpiece of its arsenal (para. 13).

So what do we do? In the end, we ask, in our pursuit to prove our *value*, who are we willing to throw away? Who are we willing to let die? Who are we willing to keep killing? Answering these questions will allow us to determine what we do next.

## Abbreviations Used

ANA: American Nurses Association
ANI: Alliance for Nursing Informatics
CMS: Centers for Medicare and Medicaid Services
HIC: Health care-industrial complex
ICN: International Council of Nurses
NCLEX: National Council Licensure Examination
NCSBN: National Council of State Boards of Nursing
NPI: National Provider Identification
PPE: Personal protective equipment
UNI: Unique nursing indicator

## References

[1] Anonymous. Dimension politique du leadership infirmier : Réflexion sur le 29e Congrès du Conseil international des infirmières et, plus largement, sur le rôle politique des organisations infirmièresPolitics of nursing leadership: Reflecting on the 29th Congress of the International Council of Nurses and on nursing organizations’ political role more broadly. Aporia 2023;15(2):3–13; doi: 10.18192/aporia.v15i2.6969

[2] ANA. Direct-Reimbursement Nursing Model. 2023. Available from: https://www.nursingworld.org/foundation/rninitiative/direct-reimbursement-nursing-model/ [Last accessed: October 2, 2023].

[3] Commission for Nurse Reimbursement. Commission for Nurse Reimbursement. 2023. Available from: https://commissionfornursereimbursement.com/ [Last accessed: October 2, 2023].

[4] Cohen J, van der Meulen Rodgers Y. The feminist political economy of Covid-19: Capitalism, women, and work. Glob Public Health 2021;16(8–9):1381–1395; doi: 10.1080/17441692.2021.1920044 33905301

[5] Adler-Bolton B, Vierkant A. Health Communism. Verso Books; 2022.

[6] Hopkins Walsh J, Dillard-Wright J. The case for “structural missingness:” A critical discourse of missed care. Nurs Philos 2020;21(1):e12279–12; doi: 10.1111/nup.12279 31583822

[7] Gustafsson N, Leino-Kilpi H, Prga I, et al. RANCARE consortium COST Action—CA15208. Missed care from the patient’s perspective—A scoping review. Patient Prefer Adherence 2020;14:383–400; doi: 10.2147/PPA.S238024 32161449 PMC7049852

[8] Jones TL, Hamilton P, Murry N. Unfinished nursing care, missed care, and implicitly rationed care: State of the science review. Int J Nurs Stud 2015;52(6):1121–1137; doi: 10.1016/j.ijnurstu.2015.02.012 25794946

[9] Sworn K, Booth A. A systematic review of the impact of ‘missed care’ in primary, community and nursing home settings. J Nurs Manag 2020;28(8):1805–1829; doi: 10.1111/jonm.12969 32017305

[10] American Nurses Association. Code of Ethics PDF. 2015. Available from: https://www.nursingworld.org/practice-policy/nursing-excellence/ethics/code-of-ethics-for-nurses/coe-view-only/ [Last accessed: Febeuary 25, 2020].

[11] Nova A. Record High Share in U.S. Report Delaying Medical Treatments Due to Costs—Here’s How You Can Save on Health Care. 2023. Available from: https://www.cnbc.com/2023/01/20/americans-put-off-health-care-because-of-cost-how-you-can-save.html [Last accessed: October 24, 2023].

[12] Butler J. Precarious Life: The Powers of Mourning and Violence. Verso; 2020.

[13] Mbembe A. Necropolitics. Duke University Press: Durham, NC; 2019.

[14] Mbembe A, Shread C. The universal right to breathe. Crit Inq 2021;47(S2):S58–S62; doi: 10.1086/711437

[15] Murray SJ. The living from the dead: disaffirming biopolitics. Penn State University Press; 2022.; doi: 10.5325/j.ctv31r2n7f

[16] Spurlin WJ. Queer theory and biomedical practice: the biomedicalization of sexuality/the cultural politics of biomedicine. In: Queer Interventions in Biomedicine and Public Health. (Garden R, Spurlin WJ. eds.) Springer International Publishing: Cham; 2023; pp. 7–20; doi: 10.1007/978-3-031-29677-2_2 PMC6373286 30073625

[17] Foucault M. Madness and Civilization. Routledge: London; 1971.; doi: 10.4324/9780203164693

[18] Foucault M. The Birth of the Clinic: An Archeology of Medical Perception. Vintage Books: New York, NY; 1994.

[19] Jacob JD, Gagnon M, McCabe J. From distress to illness: A critical analysis of medicalization and its effects in clinical practice. J Psychiatr Ment Health Nurs 2014;21(3):257–263; doi: 10.1111/jpm.12078 23638977

[20] Holmes D, Murray SJ, Perron A, et al. Deconstructing the evidence-based discourse in health sciences: Truth, power and fascism. Int J Evid Based Healthc 2006;4(3):180–186; doi: 10.1111/j.1479-6988.2006.00041.x 21631765

[21] Prescod-Weinstein C. Making black women scientists under white empiricism: The racialization of epistemology in physics. Signs J Women Cult Soc 2020;45(2):421–447; doi: 10.1086/704991

[22] Sjoding MW, Dickson RP, Iwashyna TJ, et al. Racial bias in pulse oximetry measurement. N Engl J Med 2020;383(25):2477–2478; doi: 10.1056/NEJMc2029240 33326721 PMC7808260

[23] Asad AL, Clair M. Racialized legal status as a social determinant of health. Soc Sci Med 2018;199:19–28; doi: 10.1016/j.socscimed.2017.03.010 28359580

[24] Wolstein J, Babey SH, Tan S, et al. Association of california immigrants’ avoidance of public programs due to immigration concerns with delayed access to health care. JAMA Netw Open 2022;5(12):e2246525; doi: 10.1001/jamanetworkopen.2022.46525 36512360 PMC9856315

[25] Roberts D. Torn Apart: How the Child Welfare System Destroys Black Families–and How Abolition Can Build a Safer World. Basic Books; 2022.

[26] Stone R. Pregnant women and substance use: Fear, stigma, and barriers to care. Health Justice 2015;3(1):2; doi: 10.1186/s40352-015-0015-5

[27] Fong K. Concealment and constraint: Child protective services fears and poor mothers’ institutional engagement. Soc Forces 2019;97(4):1785–1810; doi: 10.1093/sf/soy093

[28] Bernstein H, Gonzalez D, Karpman M, et al. Adults in Immigrant Families Report Avoiding Routine Activities Because of Immigration Concerns. 2019.

[29] Holmes D, Gagnon M. Power, discourse, and resistance: Poststructuralist influences in nursing. Nurs Philos 2018;19(1):1–6; doi: 10.1111/nup.12200 29143430

[30] Smith J, Willis E, Hopkins-Walsh J, et al. The Vitruvian nurse and burnout: New materialist approaches to impossible ideals. Nurs Inq 2023;31(1):e12538; doi: 10.1111/nin.12538 36424518

[31] Smith J, Klumbytė G, Sidebottom K, et al. We all care, ALL the time. Nurs Inq 2023;31(1):e12572; doi: 10.1111/nin.12572 37335684

[32] Ashley JA. Hospitals, Paternalism and the Role of the Nurse. Teachers’ College Press: New York, NY; 1976.

[33] Fraser N. Crisis of Care? On the Social-Reproductive Contradictions of Contemporary Capitalism. In: Social Reproduction Theory: Remapping Class, Recentring Oppression. (Bhattacharya T. eds.) Pluto Press: London; 2017; pp. 21–36.

[34] Weinbaum AE. Marx, Irigaray, and the Politics of Reproduction. Differences 1994;6(1):98–128; doi: 10.1215/10407391-6-1-98 12290609

[35] American Nurses Foundation. Direct-Reimbursement Nursing Model. 2022. Available from: https://www.nursingworld.org/foundation/rninitiative/direct-reimbursement-nursing-model/ [Last accessed: October 26, 2023].

[36] Gastón P. Moralizing the strike: Nurses associations and the justification of workplace conflict in california hospitals. Am J Sociol 2022;128(1):47–93; doi: 10.1086/720300

[37] Murphy MJ. Exploring the ethics of a nurses’ strike during a pandemic. Am J Nurs 2022;122(3):49–54; doi: 10.1097/01.NAJ.0000823000.39601.b1 35200190

[38] Thier H. A people’s guide to captialism: An introduction to marxist economics. Haymarket Books; 2020.

[39] Sterba S. Neoliberal Capitalism and the Evolution of the US Healthcare System. 2020

[40] McKinlay JB. Issues in the Political Economy of Healthcare. Routledge; 2022.

[41] Marx K. Capital: Volume I. Penguin books: UK; 2004.

[42] Eyer J. Capitalism, Health, and Illness. In: Issues in the Political Economy of Health Care Routledge; 2022; pp. 1–28.

[43] Klein N. The Shock Doctrine: The Rise of Disaster Capitalism. Macmillan; 2007.

[44] Dillard-Wright J. A radical imagination for nursing: Generative insurrection, creative resistance. Nurs Philos 2022;23(1):e12371; doi: 10.1111/nup.12371 34632696

[45] Berliner HS, Ginzberg E. Why this hospital nursing shortage is different. Jama 2002;288(21):2742–2744; doi: 10.1001/jama.288.21.2742 12460099

[46] Bruhn WE, Fracica EA, Makary MA. Group purchasing organizations, health care costs, and drug shortages. Jama 2018;320(18):1859–1860; doi: 10.1001/jama.2018.13604 30347037

[47] Drennan VM, Ross F. Global nurse shortages-the facts, the impact and action for change. Br Med Bull 2019;130(1):25–37; doi: 10.1093/bmb/ldz014 31086957

[48] Dyrbye LN, Shanafelt TD, Johnson PO, et al. A cross-sectional study exploring the relationship between burnout, absenteeism, and job performance among American nurses. BMC Nurs 2019;18(1):57; doi: 10.1186/s12912-019-0382-7 31768129 PMC6873742

[49] Fagin CM, Maraldo PJ. The nursing shortage. Feminism & the nursing shortage: Do women have a choice? Nurs Health Care 1988;9(7):364–367. 3173794

[50] Joel LA. Nursing shortage: A new stage of activism. Am Nurse 1989;21(3):6. 2930041

[51] Pearson A. The shortage of nurses: Is it ‘man’-made? Int J Nurs Pract 2004;10(4):143–144; doi: 10.1111/j.1440-172X.2004.00479.x 15265223

[52] Leung S. On Friday, a Round of Applause for Doctors, Nurses, Grocery Workers and Others on the Front Lines of Coronavirus—The Boston Globe. 2020. Available from: https://www.bostonglobe.com/2020/03/29/metro/friday-round-applause-doctors-nurses-grocery-workers-others-front-lines-coronavirus/ [Last accessed: October 30, 2023].

[53] Allen M. A Nurse Bought Protective Supplies for Her Colleagues Using GoFundMe. The Hospital Suspended Her. 2020. Available from: https://www.propublica.org/article/a-nurse-bought-protective-supplies-for-her-colleagues-using-gofundme-the-hospital-suspended-her [Last accessed: October 30, 2023].

[54] National Nurses United. Nurses to President Trump: Get Us Protective Equipment Now. 2020. Available from: https://www.nationalnursesunited.org/press/nurses-president-trump-get-us-protective-equipment-now [Last accessed: April 14, 2021].

[55] Beggan J, Allison S. Pressures to comply or defy: How social values influence perceptions of healthcare workers as villains. Heroism Sci 2023;8(1); doi: 10.26736/hs.2023.01.01

[56] Dreher A. Most Hospitals Profited off of Pandemic, Study Finds. 2023. Available from: https://www.axios.com/2023/07/20/hospitals-record-margins-during-pandemic [Last accessed: November 6, 2023].

[57] Einboden R. SuperNurse? Troubling the Hero Discourse in COVID Times. Health (London) 2020;24(4):343–347; doi: 10.1177/1363459320934280 32538181

[58] Holpuch A. Pandemic Profits: Top US Health Insurers Make Billions in Second Quarter. The Guardian 2021.

[59] Stokes‐Parish J, Elliott R, Rolls K, et al. Angels and heroes: The unintended consequence of the hero narrative. J Nurs Scholarsh 2020;52(5):462–466; doi: 10.1111/jnu.12591 32856396 PMC7461164

[60] Robinson CJ. Black Marxism, Revised and Updated Third Edition: The Making of the Black Radical Tradition. UNC press Books; 2020.

[61] Pirtle W. Racial capitalism: A fundamental cause of novel coronavirus (COVID-19) and pandemic inequities in the United States. Health Educ Behav 2020;47(4):504–508; doi: 10.1177/10901981209229 32338071 PMC7301291

[62] Fraser N. Expropriation and exploitation in racialized capitalism: A reply to Michael Dawson. Crit Hist Stud 2016;3(1):163–178.

[63] Dillard-Wright J, Jenkins D. Nursing as total institution. Nurs Philos 2023;25(1):e12460; doi: 10.1111/nup.12460 37403431

[64] Luxemburg R. The Complete Works of Rosa Luxemburg, Volume I: Economic Writings 1. Verso Books; 2013.

[65] Melamed J. Racial Capitalism. Crit Ethn Stud 2015;1(1):76–85; doi: 10.5749/jcritethnstud.1.1.0076

[66] Anderson WC. The Nation on No Map: Black Anarchism and Abolition. AK Press; 2021.

[67] Harris M. Kids These Days: Human Capital And The Making Of Millennials. Little, Brown and Company: New York; 2017.

[68] Reich R. When America’s Richest Men Pay $0 in Income Tax, This Is Wealth Supremacy. The Guardian 2021

[69] Braidott R. Posthuman Feminism. Polity 2021.

[70] Alexandersson A, Kalonaityte V. Girl bosses, punk poodles, and pink smoothies: Girlhood as Enterprising Femininity. Gender Work & Organization 2021;28(1):416–438; doi: 10.1111/gwao.12582

[71] Wang J. Carceral Capitalism. MIT Press; 2018.

[72] Salmon J. Oganizing Medical Care for Profit. In: Issues in the Political Economy of Healthcare. (McKinlay JB. eds.) Routledge; 2022; pp. 89–117.

[73] American Nurses Association. ANA Enterprise Leads National Efforts to Achieve Equitable Reimbursement of Nursing Care. 2023. Available from: https://www.nursingworld.org/news/news-releases/2023/anaenterprise-leads-rn-value-reimbursement/ [Last accessed: October 30, 2023].

[74] Beale N, Carroll W, Aldrich K, et al. What a Unique Nurse Identifier Means for the Future. 2021. Available from: https://www.myamericannurse.com/uni-what-a-unique-nurse-identifier-means-for-the-future/ [Last accessed: October 30, 2023].

[75] Chan GK, Cummins MR, Taylor CS, et al. An overview and policy implications of national nurse identifier systems: A call for unity and integration. Nurs Outlook 2023;71(2):101892; doi: 10.1016/j.outlook.2022.10.005 36641315

[76] HIMSS. Advocating for a Unique Nurse Identifier. 2023. Available from: https://www.himss.org/sites/hde/files/Advocating%20for%20a%20Unique%20Nurse%20Identifier.pdf [Last accessed: November 2, 2023].

[77] Beresford MJ. Medical reductionism: Lessons from the great philosophers. QJM 2010;103(9):721–724. 20395240 10.1093/qjmed/hcq057

[78] Sensmeier J, Androwich I, Baernholdt M, et al. Demonstrating the value of nursing care through use of a unique nurse identifier. - Line J Nurs Inform OJNI 2019;23(2).

[79] Lee U, Tolivar D. Building consentful tech. Ripple mapping tool. 2017. Available from: https://www.consentfultech.io/wp-content/uploads/2019/10/Building-Consentful-Tech.pdf.

[80] Beale N, Rajwany N. Implementation of a unique nurse identifier. Nurs Manage 2022;53(1):6–9; doi: 10.1097/01.NUMA.0000805040.87004.37 34979521

[81] The Invisible Committee. Now. Semiotext(e) Intervention 1. MIT Press; 2017.

[82] Duffy M. Doing the dirty work: Gender, race, and reproductive labor in historical perspective. Gend Soc 2007;21(3):313–336; doi: 10.1177/0891243207300764

[83] Derber C. Sponsorship and the control of physicians. Theor Soc 1983;12(5):561–601.

[84] Derber C. Physicians and Their Sponsors: The New Medical Relations of Production. (McKinlay JB. eds.) Routledge; 2022; pp. 141–189.

[85] Spade D. Mutual Aid: Building Solidarity during This Crisis (and the Next). Verso Books; 2020.

[86] The Invisible Committee. To Our Friends. Semiotext: South Pasadena, CA; 2015.

